# Supplementary material for: A plant cytorhabdovirus modulates locomotor activity of insect vectors to enhance virus transmission
Source: Nat Commun. 2023 Sep 16;14:5754. doi: 10.1038/s41467-023-41503-3 (PMC10505171; doi:10.1038/s41467-023-41503-3)
Supplement: Supplementary file 1 — Supplementary Information [file 41467_2023_41503_MOESM1_ESM.pdf]

Supplementary Materials for:

A plant cytorhabdovirus modulates locomotor activity of insect vectors to enhance virus transmission

Dong-Min Gao[1], Ji-Hui Qiao[1], Qiang Gao[1, 2], Jia-Wen Zhang[3], Ying Zang[1], Liang Xie[1], Yan Zhang[3], Ying Wang[2], Jingyan Fu[3], Hua Zhang[3], Cheng-Gui Han[2], Xian-Bing Wang[1]*

[1]State Key Laboratory of Plant Environmental Resilience, College of Biological Sciences, China Agricultural University, Beijing 100193, China

[2]College of Plant Protection, China Agricultural University, Beijing 100193, China

[3]State Key Laboratory of Animal Biotech Breeding, College of Biological Sciences, China Agricultural University, Beijing 100193, China

* Author for correspondence:

Xian-Bing Wang, *Email*: wangxianbing@cau.edu.cn

This PDF file includes:

Supplementary Figure 1 to 11

Supplementary Table 1

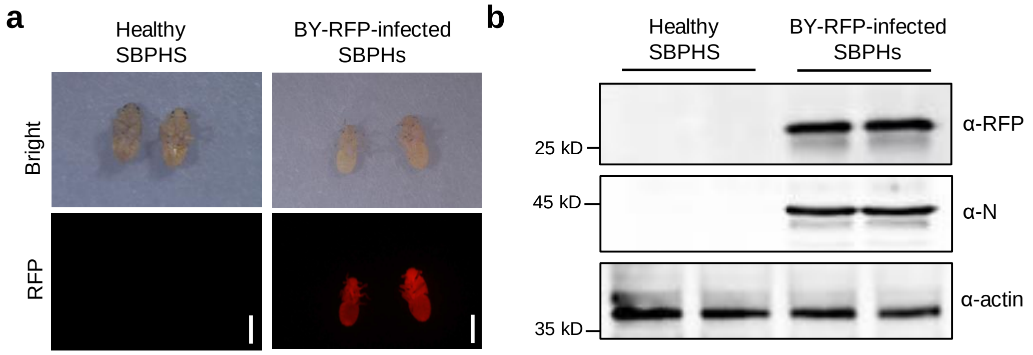

Supplementary Fig. 1. Small brown planthopper infected by BY-RFP. a RFP fluorescence of SBPHs observed with a brightfield and epifluorescence microscopy at 12 d after feeding on BY-RFP-infected barley plants. Healthy SBPHs served as negative controls. Scale bars, 2 mm. **b** Immunoblotting blotting analyses detecting accumulation of RFP and BYSMV N in SBPHs shown in panel (**a**). Actin served as loading control. All experiments were repeated three times independently with similar results. Source data are provided as the Source Data file.

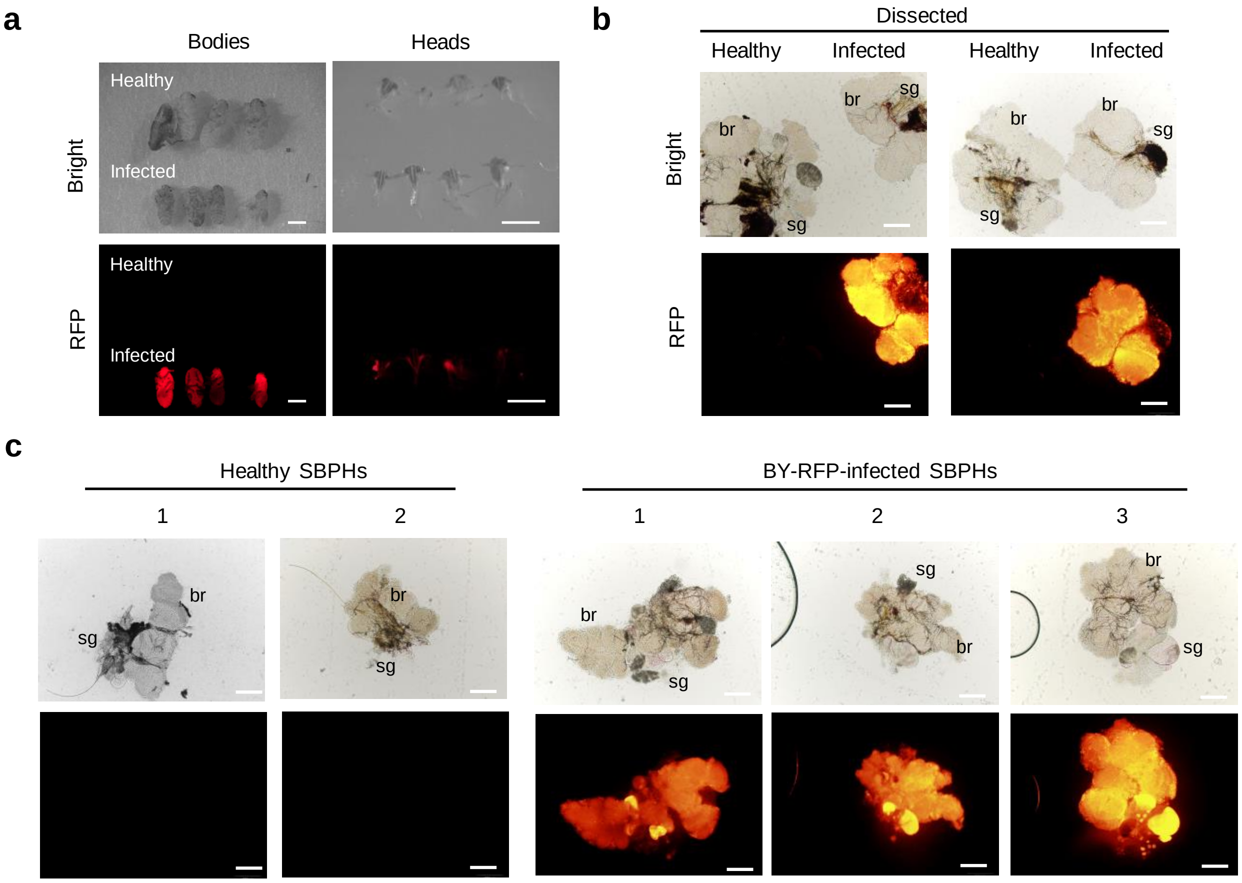

Supplementary Fig. 2. Observation of BY-RFP infection in brain tissues of viruliferous SBPHs. Healthy and BY-RFP-infected SBPHs at 15 dpi were dissected and directly observed with a brightfield and epifluorescence microscopy. **a** Whole bodies and heads of SBPHs. Scale bars, 2 mm. **b** Dissected heads were observed together un microscopy. Scale bars, 200 μm. **c** Dissected heads were observed individually with a brightfield and epifluorescence microscopy. Scale bars, 200 μm. br, brain; sg, salivary gland. All experiments were repeated three times independently with similar results.

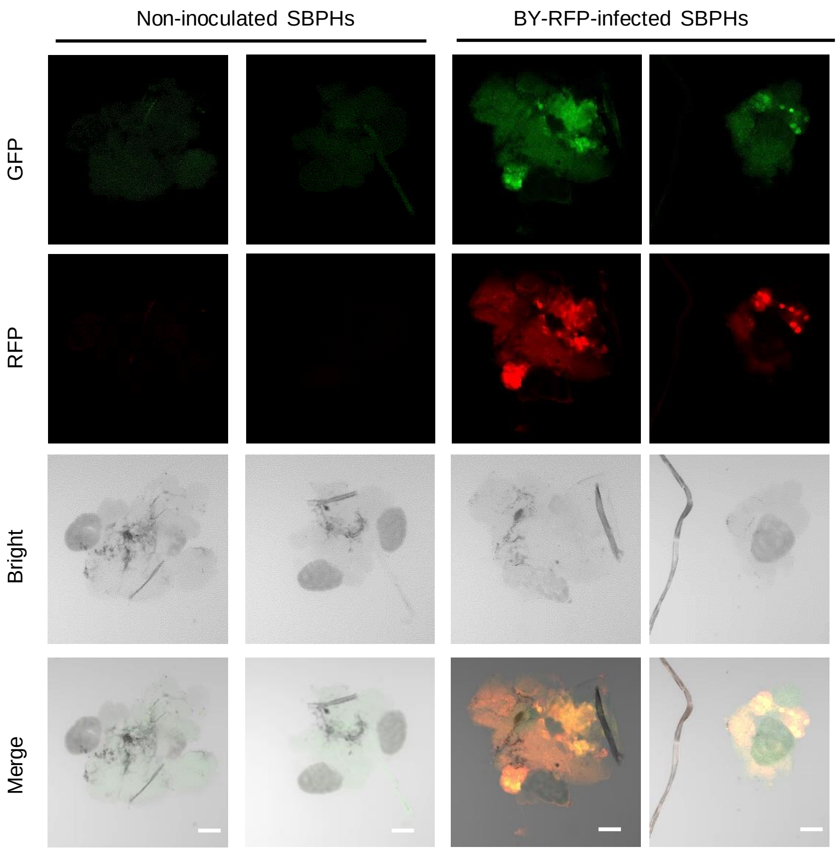

Supplementary Fig. 3. Immunological detection of N protein in brain tissues of SBPHs. Heads of non-inoculated and BY-RFP-infected SBPHs at 12 dpi were dissected and placed in 4% paraformaldehyde for fixation, followed by the added of rabbit antibodies to the N protein, and incubation with alex-488-coupled rabbit secondary antibody (GFP), observed with a brightfield and epifluorescence microscopy. Scale bars, 200 μm. All experiments were repeated three times independently with similar results.

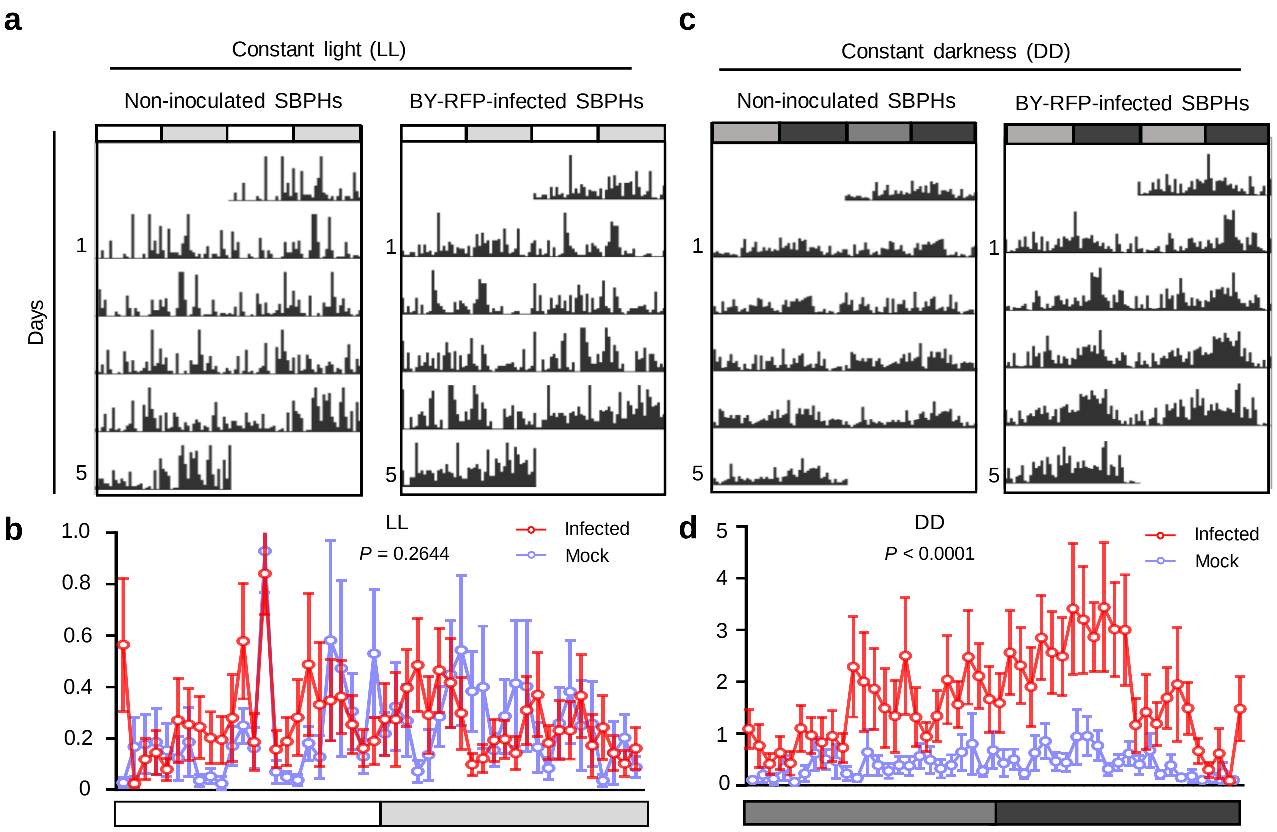

Supplementary Fig. 4. Locomotor activity analyses of SBPHs in constant light (LL) and darkness (DD). a Locomotor activity of SBPHs in constant light (LL). The column numbers represent sums of non-inoculated or BY-RFP-infected SBPHs (n = 32). **b** Locomotor activity indicated by average activity counts (y-axis) per insects for each 30 min bin in (**a**). **c** Locomotor activity of SBPHs in constant darkness (DD). The column numbers represent sums of non-inoculated or BY-RFP-infected SBPHs (n = 16). **d** Locomotor activity indicated by average activity counts (y-axis) per insects for each 30 min bin in (**a**). In (**b**) and (**d**), two-way ANOVA followed by Tukey's test was performed to investigate main effects of virus infection on insect activity. Differences were considered significant at $P < 0.05$. Source data are provided as the Source Data file.

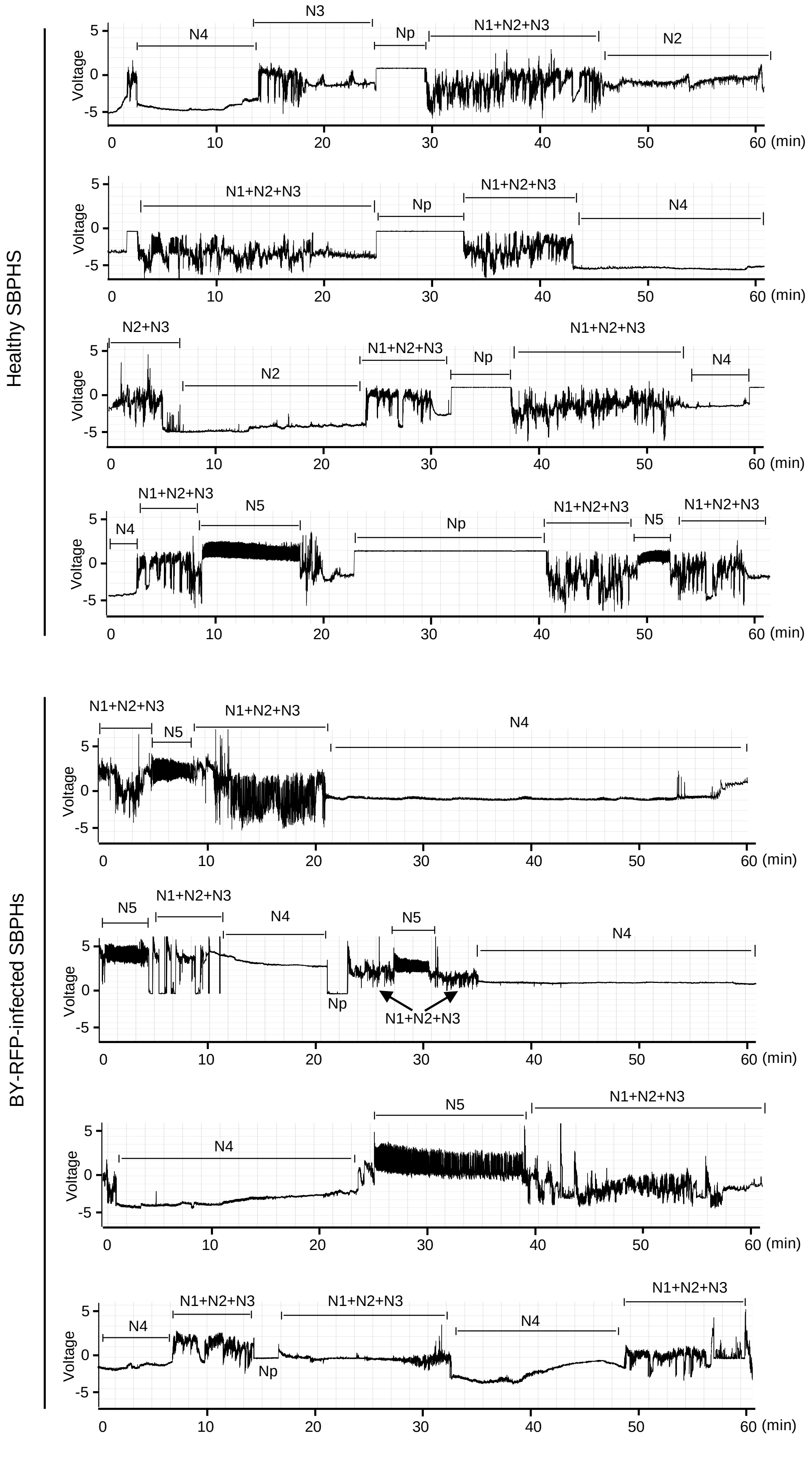

Supplementary Fig. 5. EPG waveforms of healthy SBPHs or BY-RFP-infected SBPHs feeding on barley plants. All the SBPHs were treated with 4-h starvation and then monitored continuously using an 8-channel DC-EPG device in an electrically grounded Faraday cage. All experiments were repeated six biologically independent SBPHs with similar results.

a

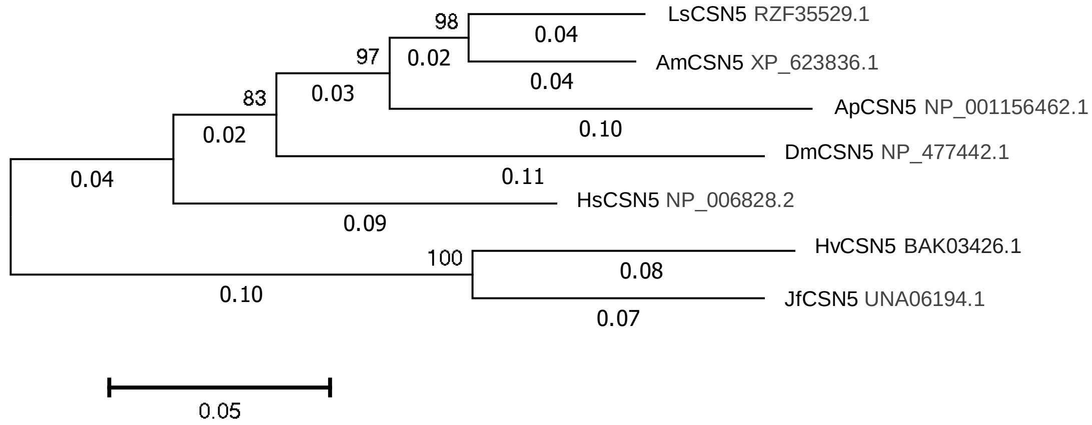

b

```
lsCSN5   MAGSSGESQSSIARKTWELSNNMETISS....VDEIYRYDRKQQQDILTAKPWEKDPHFFKDIKISALALLKMVMHARSG    76
HvCSN5   MEPTS...SSAVARQTWELENNIPAAASDPDAMDAIYRYDEAANARAHQEKPWATDPHHFRRARISALALLKMVVHARAG    77
Consensusm   s    s  ar twel nn     s     d iyryd          kpw  dph f    isalallkmv har g

lsCSN5   GTLEVMGLLLGKVDANTMIVMDSFALPVEGTETRVNAQAQAYEYMTAYIEAAKSVGRMENAIGWYHSHPGYGCWLSGIDV   156
HvCSN5   GTIEIMGLMQGKFEGDSIIVMDAFALPVEGTETRVNAQADAYEYMVEYSTINKQAGRLENVVGWYHSHPGYGCWLSGIDV   157
Consensusgt e mgl  gk      ivmd falpvegtetrvnaqa ayeym  y    k  gr en  gwyhshpgygcwlsgidv

lsCSN5   STQMLNQNFQEPFVAIVIDPVRTISAGKVCLGAFRTYPKGYKPANEEPSEYQTIPLNKIEDFGVHCKQYYSLDVTYFKSA   236
HvCSN5   STQMLNQQFQEPFLAVVIDPTRTVSAGKVDIGAFRTYPKDYKPPDEPVSEYQTIPLNKIEDFGVHCKQYYSLDITYFKSS   237
Consensusstqmlnq fqepf a vidp rt sagkv  gafrtypk ykp  e  seyqtiplnkiedfgvhckqyysld tyfks

lsCSN5   LDRRLLDSLWNKYWVNTLSSSSLITNADYTTGQIFDLSDKLEQSESSLGRGCF.MLGGTDPHDRRTEDKLLKASKDSCKT   315
HvCSN5   LDSHLLDLLWNKYWVNTLSSSPLLGNRDYVAGQIFDLADKLEQAEGQLAHSRFGMLMPSQRKKEQEESPLAKVTRDSSKI   317
Consensusld  lld lwnkywvntlsss l  n dy  gqifdl dkleq e  l    f ml          e  l k   ds k

lsCSN5   TIEIIHGLMAQMIKDRLFNHINKTSKVPV.................                                      344
HvCSN5   TAEQVHGLMSQVIKDILFNSVHPSSKSKASASGAAPDSPVPEPMVE                                      363
Consensust e  hglm q ikd lfn     sk
```

c

```
lsCSN5   MAGSSGESQSSIARKTWELSNNMETISSVDEIYRYDRKQQQDILTAKPWEKDPHFFKDIKISALALLKMVMHARSGGTLE    80
DmCSN5   .......MDSDAAQKTWELENNIQTLPSCDEIFRYDAEQQRQIIDAKPWEKDPHFFKDIKISALALLKMVMHARSGGTLE    73
Consensus         s  a ktwel nn  t  s dei ryd  qq  i  akpwekdphffkdikisalallkmvmharsggtle

lsCSN5   VMGLLLGKVDANTMIVMDSFALPVEGTETRVNAQAQAYEYMTAYIEAAKSVGRMENAIGWYHSHPGYGCWLSGIDVSTQM   160
DmCSN5   VMGLMLGKVEDNTMIVMDAFALPVEGTETRVNAQAQAYEYMTAYMEAAKEVGRMEHAVGWYHSHPGYGCWLSGIDVSTQM   153
Consensusvmgl lgkv  ntmivmd falpvegtetrvnaqaqayeymtay eaak vgrme a gwyhshpgygcwlsgidvstqm

lsCSN5   LNQNFQEPFVAIVIDPVRTISAGKVCLGAFRTYPKGYKPANEEPSEYQTIPLNKIEDFGVHCKQYYSLDVTYFKSALDRR   240
DmCSN5   LNQTYQEPFVAIVVDPVRTVSAGKVCLGAFRTYPKGYKPPNEEPSEYQTIPLNKIEDFGVHCKQYYPLEISYFKSALDRR   233
Consensuslnq  qepfvaiv dpvrt sagkvclgafrtypkgykp neepseyqtiplnkiedfgvhckqyy l   yfksaldrr

lsCSN5   LLDSLWNKYWVNTLSSSSLITNADYTTGQIFDLSDKLEQSESSLGRGCFMLGGTDPHDRRTEDKLLKASKDSCKTTIEII   320
DmCSN5   LLDSLWNKYWVNTLGSSGLLTNTEYTTGQIMDLSEKLEQSENFLGR......GTDVNEKRSEDKLSKATRDCSRSTIELI   307
Consensuslldslwnkywvntl ss l tn  yttgqi dls kleqse  lgr      gtd    r edkl ka  d    tie i

lsCSN5   HGLMAQMIKDRLFNHINKTSKVP                                                             343
DmCSN5   HGLMAQIVKDKLFNKVGLGK...                                                             327
Consensushglmaq  kd lfn
```

Supplementary Fig. 6. Phylogenetic tree and sequence alignment of CSN5 orthologues. **a** The phylogenetic tree was constructed by mega 6.0, bootstraps values indicated in each node. Accession numbers of CSN5 proteins were listed. The CSN5 orthologues are from *Laodelphax striatellus* L (ls), *Apis mellifera* (Am), *Acyrthosiphon pisum* (Ap), *Homo sapiens* (Hs), *Drosophila melanogaster* (Dm), *Hordeum vulgare L.* (Hv), and *Jacobiasca formosana* (Jf). **b** Amino acid sequence alignment of CSN5 orthologues of *Laodelphax striatellus* and barley plants. **c** Amino acid sequence alignment of CSN5 orthologues of *Laodelphax striatellus* and *Drosophila melanogaster*.

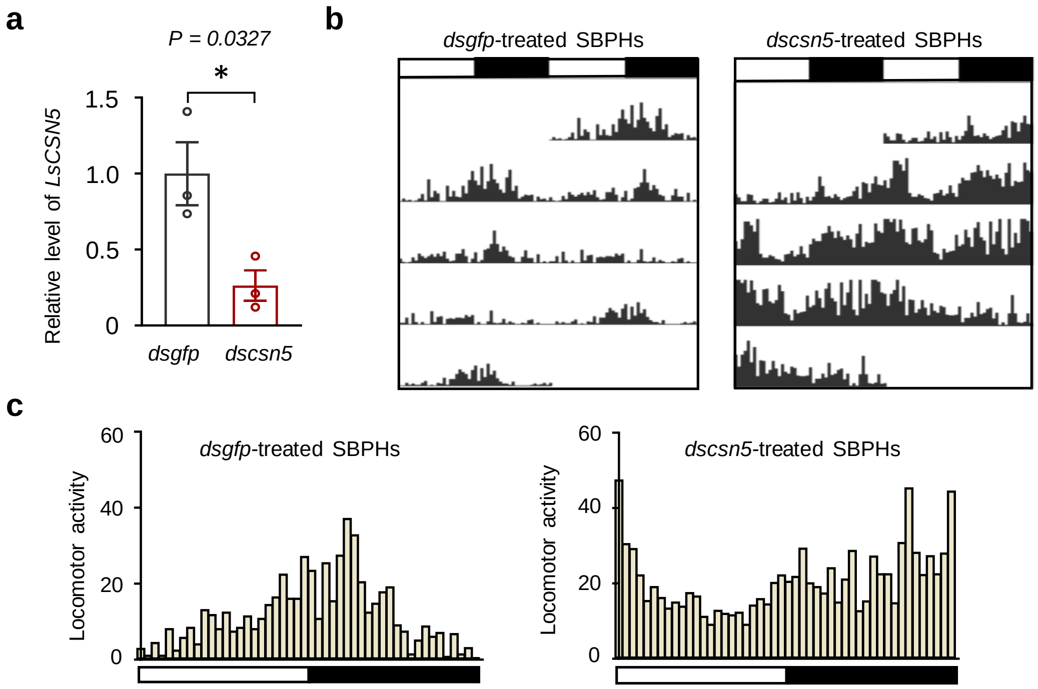

Supplementary Fig. 7. Silencing confirmation and locomotor activity of *dsgfp*- or *dscsn5*-injected SBPHs. **a** RT-qPCR detected relative accumulation of *Lscsn5* mRNA in heads dissected from *dsgfp*- or *dscsn5*-injected SBPHs. The error bars indicate SEM (n = 3 biologically independent experiments, 20 insect heads per repeat). **P* < 0.05 (two-sided t test). **b** The locomotor activity of *dsgfp*- or *dscsn5*-injected SBPHs in light/dark (LD) cycles. The column numbers represent sums of 32 SBPHs. White and black bars indicate light and dark phases, respectively. **c** Locomotor activity indicated by average activity counts (y-axis) every 30 min of SBPHs in (**b**) through 24 h, averaged over 5 LD days. Source data are provided as the Source Data file.

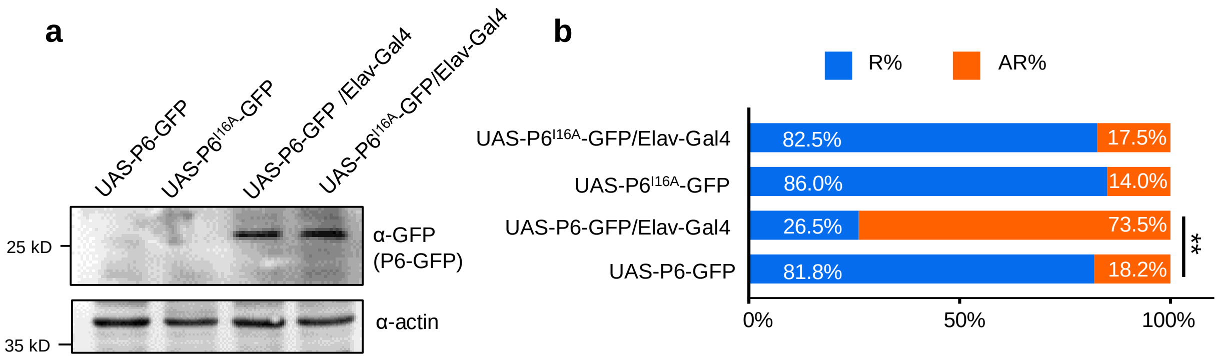

Supplementary Fig. 8. Rhythmicity and immunoblotting analysis of transgenic flies. a Immunoblotting analyses detected P6-GFP or P6^{I16A}-GFP in heads of transgenic flies. Actin served as a loading control. These experiments were repeated three times independently with similar results. **b** Heterologous expression of BYSMV P6 induced arrhythmicity in flies. Rhythmic flies were defined with the following criteria of $\chi 2$ periodogram analysis: power ≥20 and width ≥1.5. AR% and R% represent the ratios of arrhythmic and rhythmic flies, respectively. **P < 0.01 (Student's t-test). Source data are provided as the Source Data file.

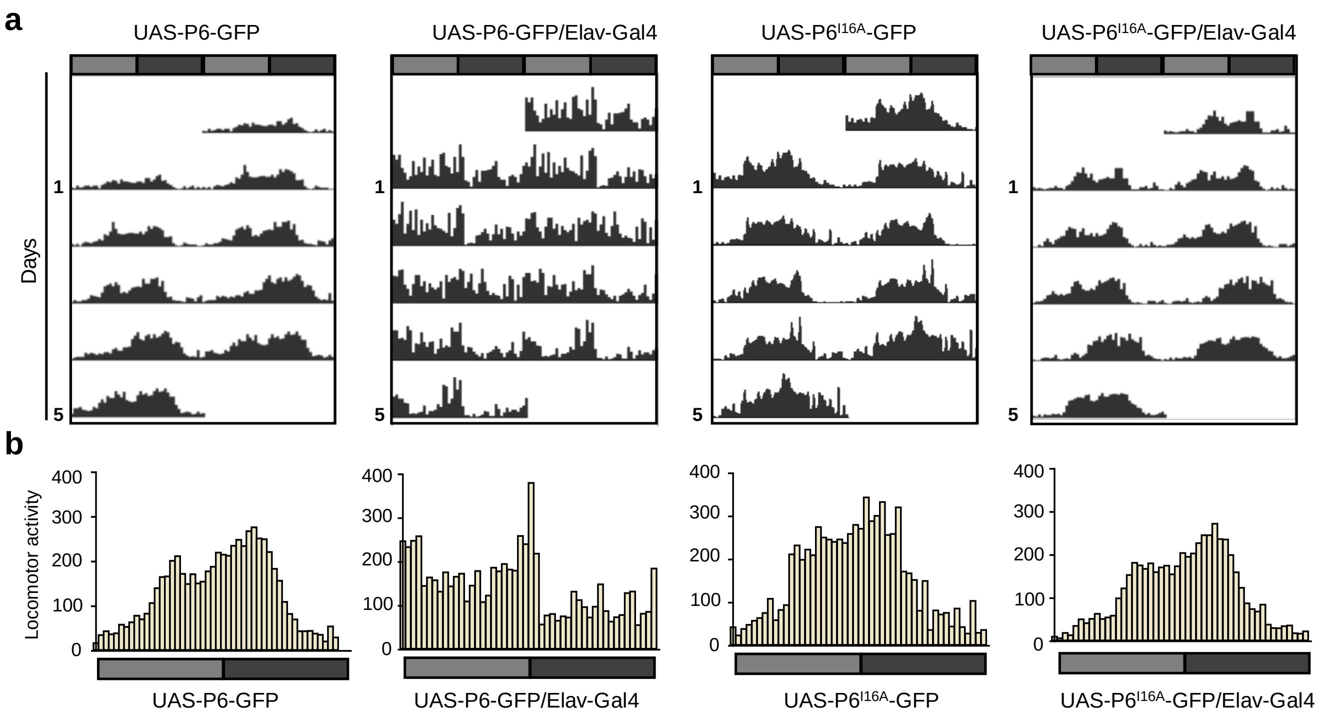

Supplementary Fig. 9. Locomotor activity analyses of transgenic flies in constant darkness (DD). a Locomotor activity of transgenic flies in DD. The fly genotypes were indicated on top of the panels. The column numbers represent sums of 16 transgenic male flies. **b** Comparison of the circadian locomotor activity of the indicated transgenic flies. Histograms represent the distribution of activity counts (y-axis) every 30 min of transgenic lines (16 male flies) through 24 h, averaged over 5 DD days. Source data are provided as the Source Data file.

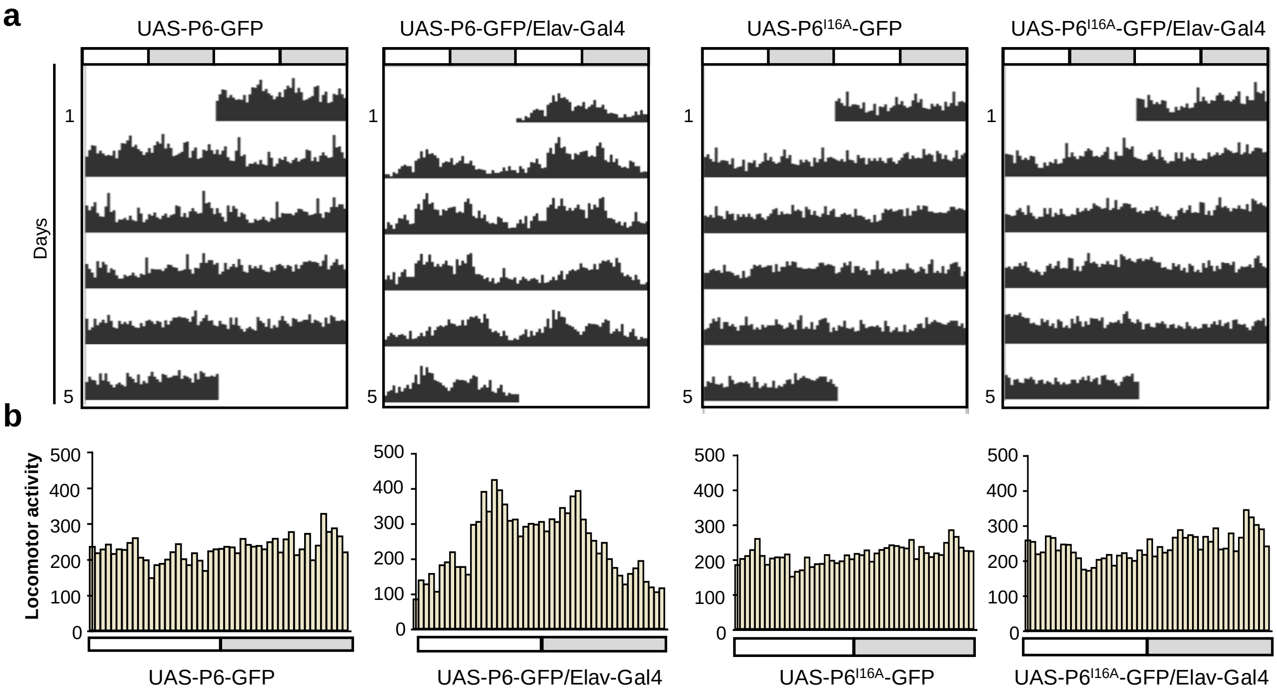

Supplementary Fig. 10. Locomotor activity analyses of transgenic flies in constant light (LL). a Locomotor activity of transgenic flies in LL. The fly genotypes were indicated on top of the panels. The column numbers represent sums of 16 transgenic male flies. **b** Comparison of the circadian locomotor activity of the indicated transgenic flies. Histograms represent the distribution of activity counts (y-axis) every 30 min of transgenic lines (16 male flies) through 24 h, averaged over 5 LL days. Source data are provided as the Source Data file.

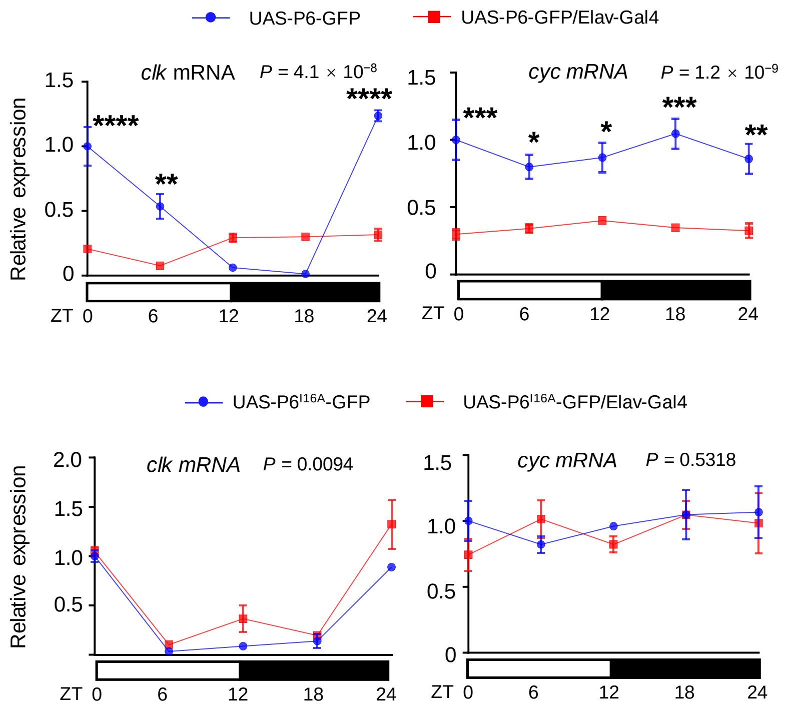

Supplementary Fig. 11. RT-qPCR analyzing relative levels of *clk* and *cyc* transcripts of dissected heads from transgenic fly lines under LD cycles. Accumulation at ZT0 of non-activated P6/P6^{I16A} (blue) was set as one unit. The *Drosophila tubulin* gene served as an endogenous control. Error bars indicate SEM of three biological independent repeats (20 insect heads per repeat). Two-way ANOVA followed by Tukey's post hoc test was performed to investigate the main effects of P6 *and* P6^{I16A} expression on mRNA levels of *tim* and *per*. Differences were considered significant at $P < 0.05$. Asterisks indicate significant differences between non-activated P6/P6^{I16A} (blue) and activated P6/P6^{I16A} (red) at the same indicated time points. *$P < 0.05$; **$P < 0.01$; ***$P < 0.001$; ****$P < 0.0001$. Source data are provided as the Source Data file.

Supplementary Table 1. List of primers used in the study.

Primer name	Sequence (5'-3')	Note
dsLsCSN5-F	TAATACGACTCACTATAGGGCATTAAAATCTCAGCACTGGCTCT	Generate lscsn5 double-stranded RNA
dsLsCSN5-R	TAATACGACTCACTATAGGGCGCCCTTGGGATAAGTCCTGA	
LsCUL1-His-F	TTAAGAAGGAGATATACAATGTACAATCTTGTAGCTCGAATCA	Generate a vector expressing LsCUL1-C-6xhis (aa 354-813)
LsCUL1-His-R	GTGGTGGTGGTGGTGCTCCGCAAGATAGCTGTAGGC	
LsTIM-N-His-F	TTAAGAAGGAGATATACAATGGAGTGGGCAGTAACTAG	Generate a vector expressing LsTIM-N-6xhis (aa 1-338)
LsTIM-N-His-R	GTGGTGGTGGTGGTGCTCGTTCTTACTACTGCCGGGG	
LsCSN5-His-F	CTTTAAGAAGGAGATATACAATGGCTGGGTCTTCGG	Generate a vector expressing LsCSN5-6xhis
LsCSN5-His-R	GTGGTGGTGGTGCTCGAGAACAGGCACCTTAGATGTC	
Dmcsn5-His-F	TTAAGAAGGAGATATACAATGGACTCCGACGCCG	Generate a vector expressing Dmcsn5-6xHis
Dmcsn5-His-R	GTGGTGGTGGTGGTGCTCTTTGCCCAGTCCGACCTT	
BK-LsCSN5- F	TGCATATGGCCATGGAGGCCATGGCTGGGTCTTCGG	Generate a vector expressing BK-LsCSN5
BK-Lscsn5- R	AGGTCGACGGATCCCCGGGTTAAACAGGCACCTTAGATGTCT	
LsperRT_F	GTCTTCAGTGGGCGACAGTT	qRT-PCR of LsPer
LsperRT_R	TGGGTCGGATGAGTCGAATG	
LstimRT_F	GCACTTGCCGTCCGATTCT	qRT-PCR of LsTim
lstimRT_R	TTTCATGGGCCCAAGCTTCT	
Lsactin_F	AAACTGGGACGACATGGAGAA	qRT-PCR of Lsactin
Lsactin_R	GCGACTCGCAACTCGTTGTA	
Lscsn5-RT-F	AATCTCAGCACTGGCTCTCC	qRT-PCR of Lscsn5
Lscsn5-RT-R	CCATTCTGCCCACACTCTT	

Dmtimeless-F	ACCCGCATCCTTCGCTTTTCTACA	qRT-PCR of DmTim
Dmtimeless-R	AGGTGAGCCAGTGGTGAGGACGGG	
Dmperiod-F	AGTTGGTCATGCGCAGCAAATG	qRT-PCR of DmPer
Dmperiod-R	TCCTTTTCGTACACAGATGCCA	
Dmtubulin-F	TCCTTGTCGCGTGTGAAACA	qRT-PCR of DmTubulin
Dmtubulin-R	CCGAACGAGTGGAAGATGAG	
Dmclock-F	TGCCCGATACGGTGGTTAT	qRT-PCR of dmclock
Dmclock-R	TTGCTGCTGGAGATTGTGC	
Dmcycle-F	TCCTGTTCGTGGTAGGTTG	qRT-PCR of dmcycle
Dmcycle-R	TGGTTGTTGCTGGCAGT	
Lsclock-F	GCACCTCCTATTATTGGCTACT	qRT-PCR of lsclock
Lsclock-R	CTGTTCTCAGAGTCAGGCATT	
Lscycle-F	GTCACAGGAGAAAGAAGCAAAC	qRT-PCR of lscycle
Lscycle-R	CGACTGATGAACTGAATGGTG	

/F: forward primer; /R: reverse primer
