# Supplementary material for: A plant cytorhabdovirus modulates locomotor activity of insect vectors to enhance virus transmission
Source: Nat Commun. 2023 Sep 16;14:5754. doi: 10.1038/s41467-023-41503-3 (PMC10505171; doi:10.1038/s41467-023-41503-3)
Supplement: Supplementary file 2 — Reporting Summary [file 41467_2023_41503_MOESM2_ESM.pdf]

nature portfolio

Corresponding author(s): Xian-Bing Wang

Last updated by author(s): Sep 6, 2023

Reporting Summary

Nature Portfolio wishes to improve the reproducibility of the work that we publish. This form provides structure for consistency and transparency in reporting. For further information on Nature Portfolio policies, see our Editorial Policies and the Editorial Policy Checklist.

Statistics

For all statistical analyses, confirm that the following items are present in the figure legend, table legend, main text, or Methods section.

n/a	Confirmed	
☐	☒	The exact sample size (n) for each experimental group/condition, given as a discrete number and unit of measurement
☐	☒	A statement on whether measurements were taken from distinct samples or whether the same sample was measured repeatedly
☐	☒	The statistical test(s) used AND whether they are one- or two-sided<br>*Only common tests should be described solely by name; describe more complex techniques in the Methods section.*
☐	☒	A description of all covariates tested
☐	☒	A description of any assumptions or corrections, such as tests of normality and adjustment for multiple comparisons
☐	☒	A full description of the statistical parameters including central tendency (e.g. means) or other basic estimates (e.g. regression coefficient) AND variation (e.g. standard deviation) or associated estimates of uncertainty (e.g. confidence intervals)
☐	☒	For null hypothesis testing, the test statistic (e.g. F, t, r) with confidence intervals, effect sizes, degrees of freedom and P value noted<br>*Give P values as exact values whenever suitable.*
☒	☐	For Bayesian analysis, information on the choice of priors and Markov chain Monte Carlo settings
☐	☒	For hierarchical and complex designs, identification of the appropriate level for tests and full reporting of outcomes
☒	☐	Estimates of effect sizes (e.g. Cohen's d, Pearson's r), indicating how they were calculated

Our web collection on statistics for biologists contains articles on many of the points above.

Software and code

Policy information about availability of computer code

Data collection	The sequences of all used genes and genomes were obtained from NCBI (https://www.ncbi.nlm.nih.gov/nuccore/). Confocal images were taken by a Leica TCS-SP8 laser scanning confocal microscope. The expression levels of mRNAs were analyzed by CFX Manger (Bio-Rad). Insect activity records were collected in 30 min bins by a Trikinetics Drosophila Activity Monitor. Signals of feeding behaviors of SPBHs on barley were monitored continuously for 8 h using an 8-channel DC-EPG device (EPG systems, Wageningen University, The Netherlands).
Data analysis	ImagJ to qualify the band intensities. Statistical analysis: Graphpad Prism8.0.2.263, Excel 2018 (Microsoft). Locomotor activity analysis: FaasX (https://neuropsi.cnrs.fr/en/departments/cnn/group-leader-francois-rouyer/) and ActogramJ (https://bene51.github.io/ActogramJ/). EPG assays analysis: stylet+ (www.epgsystems.eu). Phylogenetic tree: MEGA version 6.06. Multiple sequences alignment: DNAMAN 8.0.

For manuscripts utilizing custom algorithms or software that are central to the research but not yet described in published literature, software must be made available to editors and reviewers. We strongly encourage code deposition in a community repository (e.g. GitHub). See the Nature Portfolio guidelines for submitting code & software for further information.

Data

Policy information about availability of data

All manuscripts must include a data availability statement. This statement should provide the following information, where applicable:

- Accession codes, unique identifiers, or web links for publicly available datasets
- A description of any restrictions on data availability
- For clinical datasets or third party data, please ensure that the statement adheres to our policy

The data that supporting the findings of this study are available within the Figures and Supplementary information. The source data for Figs. 1–6, and Supplementary Figs. 1, 4, 7–11 are provided as a Source Data file. Source data are provided with this paper.

Research involving human participants, their data, or biological material

Policy information about studies with human participants or human data. See also policy information about sex, gender (identity/presentation), and sexual orientation and race, ethnicity and racism.

Reporting on sex and gender	Not applicable
Reporting on race, ethnicity, or other socially relevant groupings	Not applicable
Population characteristics	Not applicable
Recruitment	Not applicable
Ethics oversight	Not applicable

Note that full information on the approval of the study protocol must also be provided in the manuscript.

Field-specific reporting

Please select the one below that is the best fit for your research. If you are not sure, read the appropriate sections before making your selection.

☒ Life sciences ☐ Behavioural & social sciences ☐ Ecological, evolutionary & environmental sciences

For a reference copy of the document with all sections, see nature.com/documents/nr-reporting-summary-flat.pdf

Life sciences study design

All studies must disclose on these points even when the disclosure is negative.

Sample size	Samples sizes were determined based on the generation of convincing and compelling results. At least three biological replicates were performed in all experiments. The number of replicates is indicated in the figure legend and/or in the method section.
Data exclusions	No data were excluded from our analyses.
Replication	All experiments were repeated at least three times with similar results.
Randomization	Samples were allocated randomly into experimental groups
Blinding	The investigators were blinded to insect and plant infection by randomly selecting insects and plants in climate chamber for infection. The investigators were not blinded to BY-RFP-infected or healthy SBPHs in locomotor analyses because the investigators needed to determine the behavior difference between the two groups. The investigators were not blinded to other molecular testing because the investigators needed to determine appropriate samples to run in particular lanes of the gel.

Reporting for specific materials, systems and methods

We require information from authors about some types of materials, experimental systems and methods used in many studies. Here, indicate whether each material, system or method listed is relevant to your study. If you are not sure if a list item applies to your research, read the appropriate section before selecting a response.

Materials & experimental systems

n/a	Involved in the study	
☐	☒	Antibodies
☒	☐	Eukaryotic cell lines
☒	☐	Palaeontology and archaeology
☐	☒	Animals and other organisms
☒	☐	Clinical data
☒	☐	Dual use research of concern
☐	☒	Plants

Methods

n/a	Involved in the study	
☐	☒	ChIP-seq
☒	☐	Flow cytometry
☒	☐	MRI-based neuroimaging

Antibodies

Antibodies used	Rabbit polyclonal anti-FLAG, Sigma Cat. No. F7425. Mouse monoclonal anti-His, Proteintech, Cat#66005-1-lg. Rabbit polyclonal anti-GST, Abcam, Cat#ab9085. Goat anti-Mouse lgG (H+L)-HLP Conjugate, BIO-RAD, Cat#170-6516. Goat anti-Rabbit lgG (H+L)-HLP Conjugate, EASYBIO, Cat#BE0101.
Validation	The validation of RFP, GFP, BYSMV N, and ACTIN of planthopper antibodies were proved in our previous paper (Ding et al.,Plant Cell,2022, 34, 3110-3127). The antibodies of LsCUL1 and LsTIM were made and validated in this study.

Animals and other research organisms

Policy information about studies involving animals; ARRIVE guidelines recommended for reporting animal research, and Sex and Gender in Research

Laboratory animals	The small brown planthoppers, 4th instar nymph; Drosophila strain w1118, adult.
Wild animals	No wild animals were used in the study.
Reporting on sex	Not applicable
Field-collected samples	No field collected samples were used in the study.
Ethics oversight	The study did not require an ethical approval.

Note that full information on the approval of the study protocol must also be provided in the manuscript.

Dual use research of concern

Policy information about [dual use research of concern]

Hazards

Could the accidental, deliberate or reckless misuse of agents or technologies generated in the work, or the application of information presented in the manuscript, pose a threat to:

No	Yes	
☒	☐	Public health
☒	☐	National security
☒	☐	Crops and/or livestock
☒	☐	Ecosystems
☒	☐	Any other significant area

Experiments of concern

Does the work involve any of these experiments of concern:

No	Yes	
☒	☐	Demonstrate how to render a vaccine ineffective
☒	☐	Confer resistance to therapeutically useful antibiotics or antiviral agents
☒	☐	Enhance the virulence of a pathogen or render a nonpathogen virulent
☒	☐	Increase transmissibility of a pathogen
☒	☐	Alter the host range of a pathogen
☒	☐	Enable evasion of diagnostic/detection modalities
☒	☐	Enable the weaponization of a biological agent or toxin
☒	☐	Any other potentially harmful combination of experiments and agents

Plants

Seed stocks	The seeds of barley plants were collected from greenhouse and used for virus infection.
Novel plant genotypes	No novel plant genotypes were generated.
Authentication	No mutants were generated. The barley plants were infected by BYSMV virus to examine the transmission capacity of insect vectors.

ChIP-seq

Data deposition

☐ Confirm that both raw and final processed data have been deposited in a public database such as GEO.

☐ Confirm that you have deposited or provided access to graph files (e.g. BED files) for the called peaks.

Data access links<br>*May remain private before publication.*	*For "Initial submission" or "Revised version" documents, provide reviewer access links. For your "Final submission" document, provide a link to the deposited data.*
Files in database submission	*Provide a list of all files available in the database submission.*
Genome browser session<br>(e.g. UCSC)	*Provide a link to an anonymized genome browser session for "Initial submission" and "Revised version" documents only, to enable peer review. Write "no longer applicable" for "Final submission" documents.*

Methodology

Replicates	*Describe the experimental replicates, specifying number, type and replicate agreement.*
Sequencing depth	*Describe the sequencing depth for each experiment, providing the total number of reads, uniquely mapped reads, length of reads and whether they were paired- or single-end.*
Antibodies	*Describe the antibodies used for the ChIP-seq experiments; as applicable, provide supplier name, catalog number, clone name, and lot number.*

Peak calling parameters	*Specify the command line program and parameters used for read mapping and peak calling, including the ChIP, control and index files used.*
Data quality	*Describe the methods used to ensure data quality in full detail, including how many peaks are at FDR 5% and above 5-fold enrichment.*
Software	*Describe the software used to collect and analyze the ChIP-seq data. For custom code that has been deposited into a community repository, provide accession details.*
